# Physical exercise is effective for neuropsychiatric symptoms in Alzheimer's disease: a systematic review

**DOI:** 10.1590/0004-282X-ANP-2020-0284

**Published:** 2021-05-01

**Authors:** Dayanne Christine Borges Mendonça, Denise Rodrigues Fernandes, Salma Soleman Hernandez, Fernando Diákson Gontijo Soares, Karina de Figueiredo, Flávia Gomes de Melo Coelho

**Affiliations:** 1 Universidade Federal do Triângulo Mineiro Programa de Pós-Graduação em Educação Física Uberaba MG Brazil Universidade Federal do Triângulo Mineiro, Programa de Pós-Graduação em Educação Física, Uberaba MG, Brazil.; 2 Universidade Federal de Uberlândia Programa de Pós-Graduação em Ciências da Saúde Uberlândia MG Brazil Universidade Federal de Uberlândia, Programa de Pós-Graduação em Ciências da Saúde, Uberlândia MG, Brazil.; 3 Universidade do Estado de Santa Catarina Programa de Pós-Graduação em Ciências do Movimento Humano Florianópolis SC Brazil Universidade do Estado de Santa Catarina, Programa de Pós-Graduação em Ciências do Movimento Humano, Florianópolis SC, Brazil.; 4 Faculdade de Medicina Araguari MG Brazil Instituto Master Professor Antônio Carlos, Faculdade de Medicina, Araguari, MG, Brazil.

**Keywords:** Exercise, Alzheimer Disease, Behavioral Symptoms, Exercício Físico, Doença de Alzheimer, Sintomas Comportamentais

## Abstract

**Background:**

Neuropsychiatric symptoms are disorders frequently seen in Alzheimer's disease. These symptoms contribute to reduction of brain reserve capacity and, in addition, they present unfavorable implications, such as: poor prognosis for the disease, increased functional decline, increased burden on the caregiver and institutionalization. This scenario makes neuropsychiatric symptoms one of the biggest problems in Alzheimer's disease, and gives rise to a need for treatments focused on improving these symptoms. Sow progress in drug trials has led to interest in exploring non-pharmacological measures for improving the neuropsychiatric symptoms of Alzheimer's disease, such as physical exercise.

**Objective:**

To ascertain the effect of exercise on the neuropsychiatric symptoms of Alzheimer's disease and its implications.

**Methods:**

This was a systematic review of effective longitudinal research, conducted by searching for articles in the PubMed, Web of Science, CINAHL and Scopus electronic databases, from 2009 to 2019. Studies in which the sample consisted of elderly people aged 65 years old or over with a diagnosis of Alzheimer's disease were included. Initially 334 articles were identified. After exclusions, 21 articles remained to be read in full. From these, five articles fitted the eligibility criteria, and a further two articles were added through manual searches in the references of the articles found.

**Results:**

Out of the seven articles analyzed in this review, five studies revealed that physical exercise had a positive effect on the neuropsychiatric symptoms of Alzheimer's disease.

**Conclusion:**

This systematic review indicated that physical exercise is a favorable non-pharmacological means for attenuating the neuropsychiatric symptoms of elderly people with Alzheimer's disease, with special attention to aerobic exercises.

## RESUMO

**Introdução:**

Sintomas neuropsiquiátricos são desordens frequentemente observadas na Doença de Alzheimer, os quais contribuem para a diminuição da capacidade de reserva cerebral e ocasiona implicações ruins, como mau prognóstico da doença, aumento do declínio funcional, aumento da sobrecarga do cuidador e institucionalização. Esse cenário faz com que os sintomas neuropsiquiátricos se tornem um dos maiores problemas da Doença de Alzheimer, incitando, assim, o interesse em explorar medidas não-farmacológicas nos sintomas neuropsiquiátricos na Doença de Alzheimer, como o exercício físico.

**Objetivo:**

Verificar o efeito do exercício físico em sintomas neuropsiquiátricos da Doença de Alzheimer e suas implicações.

**Métodos:**

Trata-se de uma revisão sistemática com pesquisas longitudinais efetivadas por meio da busca de artigos nas bases de dados eletrônicas: PubMed, Web of Science, CINAHL e Scopus, de 2009 a 2019. Foram incluídos estudos cuja amostra foi constituída por idosos com diagnóstico de Doença de Alzheimer com idade igual ou acima de 65 anos. Inicialmente, foram identificados 334 artigos; após as exclusões, restaram 21 artigos para leitura na íntegra. Destes, cinco artigos se enquadraram nos critérios de elegibilidade, assim como dois artigos adicionados por meio de busca manual nas referências dos artigos encontrados.

**Resultados:**

Dos sete artigos analisados na presente revisão, cinco estudos revelaram um efeito positivo do exercício físico em sintomas neuropsiquiátricos na Doença de Alzheimer.

**Conclusão:**

Esta revisão sistemática indica que o exercício físico é um meio não-farmacológico favorável à atenuação dos sintomas neuropsiquiátricos de idosos com Doença de Alzheimer, com especial atenção na modalidade aeróbia.

## INTRODUCTION

Neuropsychiatric symptoms (NPS) are frequently present in Alzheimer's disease (AD). They contribute to reduction of brain reserve capacity and have unfavorable implications, such as: poor prognosis for the disease, increased burden on the caregiver, increased expenditure on care for patients with AD, increased functional decline and institutionalization^1^^,^^2^. This scenario makes NPS one of the biggest problems in AD, and gives rise to a need for treatments focused on improving NPS. The most common symptoms are apathy, depression, sleep disturbance, anxiety, irritability, agitation, euphoria, disinhibition, changes in appetite, delusions and hallucinations^3^^,^^4^.

Currently, the main form of therapy for these symptoms consists of use of medications. Antidepressants, anticonvulsants, typical and atypical antipsychotics and mood stabilizers have been used among patients with dementia. However, some harmful effects that trigger medical complications are often associated with use of these medications^5^. Clinical trials on the use of antipsychotics in AD have demonstrated that these drugs have minimal effects in comparison with placebo, in addition to a higher risk of mortality (between 60 and 70%), compared with groups that received placebo^6^^,^^7^.

Slow progress in drug trials has led to interest in exploring non-pharmacological measures for NPS in AD, such as physical exercise. In a systematic review that aimed to compare studies addressing the effectiveness of drug therapy and physical exercise for diminishing the impairment of people with AD and mild cognitive deficits pointed out that interventions through physical exercise have more potential for improving cognition in AD than medications, which had as mall effect size^8^. In another study carried out among 200 participants with mild AD, a group that underwent a physical exercise program was compared with a control group, and it was demonstrated that the group that practiced exercises significantly reduced its NPS^9^.

Given the increasing numbers of elderly people with AD in Brazil and the presence of NPS in the majority of these people, it is of the utmost importance to ascertain the effects of physical exercise regarding NPS in AD and the implications of this, by means of an investigation of the literature. Moreover, there is now a notable need for new therapeutic strategies in order to mitigate these symptoms and consequently contribute to improvement of the conditions and social attributes of AD.

Through the present study, we intend not only to discuss the effectiveness of a physical exercise proposal for NPS in AD, but also to identify parameters for better prescription of a systematic accompanied physical exercise proposal. Therefore, we would expect that this study can mediate a conceptual basis for proposing new perspectives in the field of assessment, prescription and monitoring of physical exercise among patients with AD and NPS. Furthermore, this may develop and encourage healthcare professionals, research and teaching, while seeking better quality of life for family members and patients diagnosed with NPS.

## METHODS

This was a systematic review about the effects of physical exercise on neuropsychiatric symptoms in AD. The present study was registered under CRD42020147841 in the International Prospective Register of Systematic Reviews (PROSPERO). It followed the recommendations of the PRISMA method (Preferred Reporting Items for Systematic reviews and Meta-Analyses) and was carried out by two independent researchers.

### Eligibility criteria

The studies that were eligible for inclusion were articles published in Brazil and elsewhere between 2009 and 2019, in longitudinal studies with samples formed by elderly people who had been diagnosed with AD (either randomized or non-randomized). In these, the dependent variable would be neuropsychiatric symptoms and the independent variables would be physical exercise performed both in the community and at home, by men and women. The following were excluded: animal studies, cross-sectional studies, reviews and meta-analyses, studies in which the sample was heterogeneous (other types of dementia or mild cognitive impairment), and studies in which the sample was less than 65 years old.

### Search strategies

This systematic review was carried out in the following electronic databases: National Library of Medicine (PubMed), Web of Science, Cumulative Index to Nursing and Allied Health Literature (CINAHL) and Scopus. The search was carried out on November 1, 2019.

MeSH terms and Boolean operators were used in the search strategy, as follows: “exercise” (MeSH) OR “exercises” OR “physical exercise” OR “physical exercises” OR “motor activity” (MeSH) OR “motor activities” OR “physical activity” OR “physical activities” OR “motor intervention” OR “physical fitness” (MeSH) OR “fitness, physical” AND “Alzheimer disease” (MeSH) OR “Alzheimer's disease” OR “Alzheimer” OR “Alzheimer's dementia” OR “disease, Alzheimer” OR “disease, Alzheimer's” OR “dementia, Alzheimer type” AND “Neuropsychiatric disturbances” OR “Neuropsychiatric symptoms” OR “Behavioral symptom” (MeSH) OR “Behavioral symptoms” OR “Problem Behavior” (MeSH) OR “Problem Behaviors” OR “Behavioral Problem” OR “Behavioral Problems” OR “Disruptive behavior” OR “Disruptive Behaviors” OR “Mental disorders” (MeSH) OR “Mental disorder”. To store and analyze the study data, the Zotero software was used, and also the Excel software to extract the selected data.

### Study selection and data extraction

The study selection and data extraction were performed by two reviewers independently. The researchers confirmed the results to each other and any differences were resolved through careful examination and the common sense of both of them. In the first instance, articles were excluded by reading the titles, and then the abstracts were analyzed. If these were not in accordance with the eligibility criteria, the articles were excluded. In the third stage, the articles were read in full, and selections were made after careful analysis. Lastly, manual searches of the references of these studies were conducted, in order to find any additional articles that might fit the criteria of this review. Afterwards, a detailed analysis of each study was carried out.

The evaluation regarding the methodological quality of the studies and the risk of bias was carried out based on the Cochrane Collaboration too land from selection of important points for good methodological quality relating to the subject in question. Therefore, the following propositions were considered: randomization of the sample (or lack of this); generation of the random sequence; evaluators’ blinding; similar groups in the initial evaluation; participants’ inclusion criteria; description of the experimental protocol; statistical comparison among groups; description of the sample losses; and description of the results.

### Analysis of the effect size of the studies selected

The effect size of the studies selected was calculated using Hedges’ g, in which: g=M1 - M2/standard deviation (difference among the means, divided by the standard deviation, in each group). Hedges’ g calculates the weighted effect size according to the relative size in each sample. The effect size analysis results in scores with small (g=0.2 to 0.4), medium (g=0.5 to 0.7) and high (g=0.8 to 2.0) equivalence. Studies that did not show average and standard deviation data for calculating the effect size were excluded.

## RESULTS

### Study description

**Figure 1:**
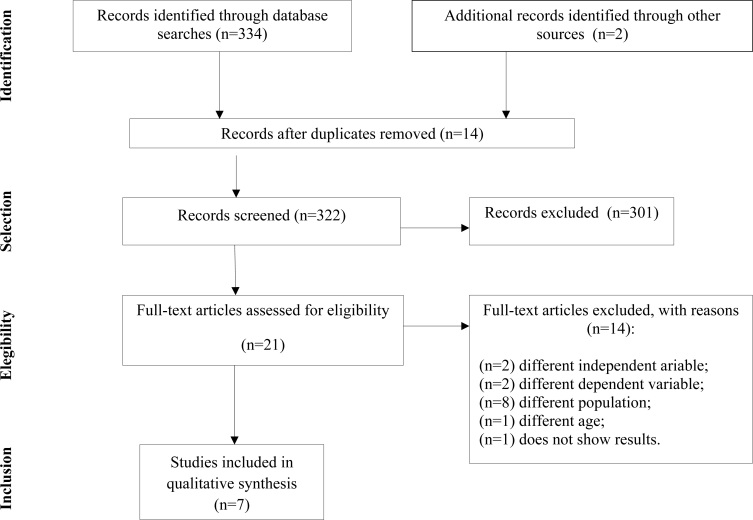
Flow diagram of the articles found in this systematic review.

Initially, 334 articles were identified. After the exclusions that were made through reading titles and abstracts and through removing duplicates, 21 articles remained for full reading. Out of these, five articles that fitted the search eligibility criteria were included, along with another two articles that were added through manual searches in the references of the articles found. Figure 1 shows the analysis flowchart of the systematic review.

### Characteristics of the studies

The characteristics of the articles included are presented in Table 1 and Table 2.

**Table 1:** Characteristics of the studies included in the review.

Author	Sample (N)	Intervention	Duration, frequency, exercise intensity
Steinberg et al.^10^	IG=14; CG=17 Community elderly Did not report stage	Multimodal exercise (aerobic fitness, strength training, balance and flexibility training)	3 months Did not have a determined duration. Every day Moderate intensity
Stella et al.^11^	EG=16; CG=16 Community elderly Mild or moderate stage	Aerobic exercise (walking, dancing and upper and lower-limb mobility)	6 months 60 minutes 3 times a week Moderate intensity
McCurryet al.^12^	WG=27; LG=25; WLG=27; CG=29 Community elderly Did not report stage	Aerobic exercise (walking)	6 months 30 minutes Every day Did not report intensity
Nascimento et al.^13^	EG=10; CG=10 Community elderly Mild or moderate stage	Multimodal exercise along with cognitive stimulation	6 months 60 minutes 3 times a week Moderate intensity
Yu et al.^14^	EG=11 Community elderly Mild or moderate stage	Aerobic exercise (cycling)	6 months 45 minutes 3 times a week Moderate intensity
Venturelli et al.^15^	AE=20; CT=20; AE+CT=20; NT=20 Institutionalized elderly Did not report stage	Aerobic exercise (walking), along with cognitive stimulation	3 months 60 minutes 5 times a week Moderate intensity
Ohman et al.^16^	GD=70; HE=70; CG=70 Community elderly Mild to severe stage	Multimodal exercise, along with cognitive stimulation	12 months 60 minutes 2 times a week Did not report intensity

EG: exercise group; CG: control group; EG: experimental group; AE: aerobic exercise; CT: cognitive training; NT: no treatment; WG: walking group; LG: light exposure group; WLG: walking, light exposure and sleep education group; HE: home-based exercise; GC: group-based exercise in day care center; IG: intervention group.

**Table 2:** Instruments used and results.

Author	Intervention	Instruments	Outcomes	Effect of exercise on NPS	Effect size
Steinberg et al.^10^	Multimodal exercise	NPI, Cornell scale	NPS, depression	Negative	–
Stella et al.^11^	Aerobic exercise	NPI, Cornell scale	NPS, depression	Positive	1.4
McCurry et al.^12^	Aerobic exercise	Actigraphy, SDI	Sleep	Positive	WG: 0.6; LG: 1.9; WLG: 1.9
Nascimento et al.^13^	Multimodal exercise	NPI	NPS	Positive	*−0.4
Yu et al.^14^	Aerobic exercise	GDS	Depression	Positive	*0.6
Venturelli et al.^15^	Aerobic exercise	NPI, ABS	NPS, agitation	Positive	–
Ohman et al.^16^	Multimodal exercise	NPI, Cornell scale	NPS, depression	Negative	–

NPI: neuropsychiatric inventory; ABS: agitated behavior scale; GDS: geriatric depression scale; NPS: neuropsychiatric symptoms; SDI: sleep disorder inventory; WG: walking group; LG: light exposure group; WLG: walking, light exposure and sleep education group.

* Value illustrated by the author himself, based on an experimental intragroup measurement.

### Evaluation of the methodological quality of the included research

The methodological quality assessment is presented in Table 3.

**Table 3:** Evaluation of the methodological quality of the studies included.

	Steinberg et al.^10^	Stella et al.^11^	McCurryet al.^12^	Nascimento et al.^13^	Yu et al.^14^	Venturelli et al.^15^	Ohman et al.^16^	Statistical analysis according to assessment item n/N(%)
Sample randomization	No	No	Yes	No	No	Yes	Yes	3/7(42.8)
Random sequence generation	No	No	Yes	No	No	No	Yes	2/7 (28.5)
Blinding of evaluators	No	No	Yes	Yes	No	Yes	No	3/7(42.8)
Similar groups in the initial assessment	No	Yes	Yes	Yes	No	Yes	Yes	5/7(71.4)
Inclusion criteria for participants	Yes	Yes	Yes	Yes	Yes	Yes	Yes	7/7(100)
Description of the experimental protocol	Yes	Yes	Yes	Yes	Yes	Yes	Yes	7/7(100)
Statistical comparison among groups	Yes	Yes	Yes	Yes	No	Yes	Yes	6/7(85.7)
Description of sample losses	Yes	Yes	Yes	Yes	Yes	Yes	Yes	7/7(100)
Results description	Yes	Yes	Yes	Yes	Yes	Yes	Yes	7/7(100)

A percentage analysis was performed to assess the risk of bias in the articles, in according with the criteria previously mentioned. The presence of these criteria is important, as they contribute to ensuring that studies are less exposed to bias or systematic errors that might compromise the accuracy of the scientific evidence.

Regarding randomization of the sample, this important methodological procedure was carried out in 42.8% of the articles. Data on generation of random sequences was presented in 28.5% of the studies. Blinding of the evaluators was described in 42.8% of the articles analyzed. In 71.4% of the articles, it was demonstrated that the groups were similar at the starting point for the intervention, confirmed through statistical comparison among the groups. All the articles presented and described the inclusion criteria for the participants, a description of the experimental protocol, a description of sample losses and a description of the results.

Thus, it was observed that out of the nine criteria evaluated in the studies, there was low adherence in relation to three criteria (randomization, generation of random sequence and evaluators’ blinding). However, the other six items analyzed were presented. Therefore, overall, good methodological quality was observed in most of the articles selected for this review, thus reducing the risk of bias.

## DISCUSSION

Over the ten-year period analyzed in the literature, seven articles that investigated the effect of physical exercise on NPS were identified. Out of these seven articles analyzed^10^^,^^11^^,^^12^^,^^13^^,^^14^^,^^15^^,^^16^, five showed that physical exercise had a positive effect on NPS, regarding Alzheimer's disease, and only two articles did not show any benefits^10^^,^^16^. However, these two studies did not evaluate NPS as a primary outcome. The main objective of these articles was to investigate the effectiveness of physical exercise on motor functionality^10^^,^^16^. From these findings, it can be inferred that physical exercise provides benefits for NPS in elderly people with AD. The analysis on the effect size showed that there were large-magnitude effects in favor of aerobic exercise protocols that involved walking and those that combined walking with light exposure and sleep education (especially concerning sleep disorders). Multimodal experimental protocols seemed not to be efficient for NPS. However, these data should be used carefully because there is not a vast amount of research on this subject and limitations were observed in the studies.

### Acute variables of the sample and physical exercise program: mode, duration, frequency and intensity

In relation to the disease stages of the populations in the studies analyzed, three studies were carried out among elderly people who were in the mild or moderate stage of the disease, and positive effects from physical exercise on NPS in AD^11^^,^^13^^,^^14^ were identified. One study was composed of elderly people who were in stages from mild to severe, in which no favorable results from physical exercise were observed with regard to NPS in AD^16^. The other studies did not specify the stage of AD that the elderly subjects were in^10^^,^^12^^,^^15^. It is important to bear in mind that the severity level of AD may influence the results from studies. It would be difficult to achieve the same intervention with older people in various stages of the disease, because of their distinct motor, cognitive, behavioral and psychologicalcharacteristics^17^.

From the interrelationship between cognitive impairment and the severity of NPS^18^ it could be seen that in the mild phase of AD, there was greater ease of learning, understanding and execution of the exercises, as well as better permanence in them, compared with what was observed among the elderly people in the moderate and advanced stage of the disease. Consequently, more favorable results can be seen in the mild phase of Alzheimer's disease with regard to practicing physical exercise.

Based on this context, the importance of professional care in the preparation for classes is emphasized. Exercises should be introducedted in an adapted and gradual way, with regard to the difficulty presented, taking the characteristics of each stage of AD into consideration. It is also essential to point out the importance of starting physical exercise in the mild phase of the disease in order to favor attenuation of AD symptoms and to postpone their evolution from one phase to another. In addition, it is relevant to highlight that besides the necessary efficiency of the professionals’ performance, in order to achieve effectiveness of physical training and adherence of the elderly subjects to the proposed activities, it is fundamental to have support and availability from family members or caregivers for encouraging regular practicing of physical activity, as also to have encouragement through public policies, including strategies, projects and guidelines regarding physical exercise aimed at the population with AD.

In general, the samples in other studies were composed of elderly people with AD who were living in the community. Only Venturelli et al.^15^ implemented an intervention among institutionalized elderly people. Their study showed a positive result regarding the influence of a walking protocol in relation to behavioral disorders among elderly people with AD. There was a significant decrease in NPS and, specifically, with regard to agitation. It has been observed that institutionalized elderly people usually have higher intensity and frequency of NPS, given that it is known that this is one of the factors that lead elderly people with AD to become institutionalized^17^. Therefore, studies in this type of environment are particularly important for enabling mitigation of these symptoms.

Among the studies conducted among non-institutionalized elderly people with AD, regarding the place where the interventions were implemented, three studies had groups that trained in their respective homes^10^^,^^12^^,^^16^. Out of these, only one group showed a reduction in NPS, specifically, at sleep time^12^. On the other hand, among the four studies^11^^,^^13^^,^^14^^,^^16^, that had groups of elderly people who trained at research centers or in specific places where these elderly subjects spent the day, three groups showed favorable responses, with decreases in NPS. These results can be attributed to the socialization factor, which added to the effectiveness of physical exercise in relation to NPS^11^^,^^13^^,^^14^.

Among the articles included in this review, five articles analyzed the effect of physical exercise in isolation, as an intervention^10^^,^^11^^,^^12^^,^^14^^,^^15^. The other two articles analyzed physical exercise combined with targeted cognitive stimulation, i.e. a motor gesture together with a predetermined cognitive task, called dual tasking^13^^,^^16^. Moreover, Venturelli et al.^15^ not only evaluated a group of elderly people who underwent physical exercise alone, but also analyzed another group that participated in physical training combined with targeted cognitive stimulation.

The five studies with groups of elderly people who solely underwent physical training used exercises of aerobic and multimodal types^10^^,^^11^^,^^12^^,^^14^^,^^15^. The three groups with dual tasking in their interventions used physical training, multimodal exercises and walking^13^^,^^15^^,^^16^. Out of the three groups of elderly people with AD who underwent dual task training, two groups achieved benefits with regard to NPS^13^^,^^15^. Among the five groups of elderly people with AD whose intervention consisted of physical exercise along (only a guided motor task), a positive effect was seen in four of the groups, with regard to the NPS outcome. In short, both the protocols for physical exercise alone and the dual task protocols proved to be efficient for attenuation of NPS. Table 4 illustrates the comparison between physical training alone and the dual task protocol.

**Table 4:** Comparison of the effect of the physical exercise protocol in isolation and the double task protocol on the neuropsychiatric symptoms of the studies.

Author	Type of intervention	Effect of the protocol on NPS
Steinberg et al.^10^	Physical exercise	Negative
Stella et al.^11^	Physical exercise	Positive
McCurry et al.^12^	Physical exercise	Positive
Nascimento et al.^13^	Dual task	Positive
Yu et al.^14^	Physical exercise	Positive
Venturelli et al.^15^	Physical exercise Dual task	Positive Positive
Ohman et al.^16^	Dual task	Negative

The protocols for multimodal exercises that were used in the interventions of three articles consisted of developing functional capacity skills such as: aerobic fitness, strength, flexibility, agility and balance^10^^,^^11^^,^^13^^,^^16^. Use of this type of protocol corroborates the findings from the systematic review by Hernandez et al.^18^, who highlighted the multimodal protocol as the most beneficial form of exercise for elderly people with AD. The other four articles presented aerobic exercise as an intervention^11^^,^^12^^,^^14^^,^^15^. These aerobic exercises involved walking, dancing, upper and lower limb movements and use of cycle ergometers. Out of the three studies that presented multimodal exercise^10^^,^^13^^,^^16^, two of them revealed a negative outcome in relation to neuropsychiatric symptoms^10^^,^^16^ and one study demonstrated a positive effect on the association between exercise and NPS^13^. The four studies in which aerobic exercises were chosen as an intervention had positive results regarding NPS. These results corroborated those of a meta-analysis carried out by Panza et al.^19^, in which they investigated 19 studies and reported that aerobic exercises had a favorable effect in relation to AD.

From the results presented in the studies included in this review, it might be inferred that aerobic exercises were the most advantageous in relation to NPS in elderly people with AD. However, caution is necessary, since the number of studies showed that analysis on this topic remains limited, with regard both to aerobic exercises and to multimodal exercises. On the other hand, this review did not find any intervention presenting the effectiveness of resistance exercises on NPS in AD.

Regarding the duration of interventions, these ranged from three months to twelve months, which are promising lengths of time for investigating the effect of physical exercise on NPS in Alzheimer's disease. It is known that physical exercise performed regularly and persistently for a long term gives rise to positive morphological and functional changes to the body. The frequency of application of the exercises described in the studies was from two to five times a week. The classes conducted in these studies lasted from 30 to 60 minutes, except in the case of Steinberg et al.^10^, who did not specify the time. The exercise intensity recommended in most articles was moderate, thus corroborating the findings of the systematic review by Hernandez et al.^18^, who investigated 12 articles and concluded that the intensity of exercise that brought the greatest benefits for elderly people with AD was moderate. This is also the intensity recommended for the elderly population according to the ACSM (American College of Sports Medicine). Only the studies by McCurry et al.^12^ and Ohman et al.^16^ did not report any specific training intensity. In this light, it is important to highlight the efficiency of prescription of physical exercises for elderly people with AD: moderate intensity of exercise provided benefits both for these individuals’ NPS, and for their motor and cognitive functioning^19^.

It is also important to report that the studies by Stella et al.^11^, Nascimento et al.^13^, Ohman et al.^16^ and Yu et al.^14^ pointed out that there was a gradual progression of intensity or load in training, which contributed to enhancement of the benefits from physical exercise. This has already been well explained in the scientific literature on the principles of physical training^20^. The participants in these studies were guided by professionals such as physical therapists and physical education professionals, which facilitated better design of the training, given that these professionals are qualified for this. Steinberg et al.^10^ used a physical exercise program in their intervention that had been developed and tested by a physiologist, but for which the caregivers of the elderly people with AD were trained. In the studies by McCurry et al.^12^ and Venturelli et al.^15^, systematized physical activity was applied by caregivers, with support and guidance from the researchers involved.

### Instruments used and outcomes

In this systematic review, the Neuropsychiatric Inventory (NPI) was identified as the test most used for analyzing NPS in research. The NPI is a scale implemented by the caregiver. It has twelve specific domains assessing the patient's behavior: hallucinations, delusions, agitation, depressive symptoms, anxiety, euphoria, apathy, disinhibition, irritability, aberrant motor behavior, night disturbances and changes of appetite. The test indicates the frequency and intensity of these behavioral disorders, which are characteristic of dementia. This instrument has good reliability and the higher the scores are, the greater the neuropsychiatric disorders also are. Its use was investigated in five articles^10^^,^^11^^,^^13^^,^^15^^,^^16^.

Among the five articles that evaluated NPS through the NPI, three articles^10^^,^^11^^,^^16^ used the Cornell depression scale concurrently. This scale is, specific for evaluating depressive symptoms in people with dementia. One article used the agitation behavior scale (ABS) to analyze agitation disorders^15^. The two studies that did not use the NPI used, respectively, actigraphy to measure the sleep-wake state; the sleep disorder inventory (SDI), a questionnaire that evaluates the intensity and frequency of behaviors relating to the caregiver's sleep and state of tiredness; and the geriatric depression scale (GDS), which is used to detect depressive symptoms in the elderly^12^^,^^14^.

In general, the articles investigated the effect of physical exercise on the twelve NPS of the NPI. The following specific symptoms were analyzed: depression, agitation and sleep disorders. These are important outcomes for investigation, since they are among the most common symptoms of AD^3^^,^^4^. Symptoms of depression in isolation were found in four articles^10^^,^^11^^,^^14^^,^^16^, and a beneficial effect was found in three of these studies. Sleep and agitation disorders were presented respectively in the studies by McCurry et al.^12^ and Venturelli et al.^15^, as specific outcomes. Improvements in these symptoms were observed among the elderly people with AD.

It is also interesting to highlight an important event that Venturelli et al.^15^ pointed out in their study: the prevalence of NPS in the early evening, in comparison with the prevalence at dawn. Even though success in mitigating NPS in two periods after the intervention was reported, it was found that the improvements in NPS through the intervention were more significant in the early evenings than at dawn. This observation that there is higher frequency of NPS in the sunset period in patients with AD, also called sundown syndrome, was confirmed through a cross-sectional study by Menegardo et al.^20^, who concluded that NPS are exacerbated at dusk, among elderly people with AD.

### Neurobiological mechanisms for physical exercise relating to neuropsychiatric symptoms of Alzheimer's disease

The main neurobiological explanations for NPS in AD involved is figurement of the frontal-subcorticalcircuits, cortical-cortical networks and monoaminergic system, which mediating human social behavior, motivation and memory-emotion^21^.

One of the mechanisms through which physical exercise helps in attenuating NPS consists of promotion of increased cerebral blood flow during physical activity and even at rest. As a result, there is greater oxygen uptake and higher glucose levels in the brain. Moreover, the release of neurotransmitters such as serotonin and dopamine, which have functions relating to behavior, mood, anxiety and depression^21^^,^^22^ also increases.

Besides contributing to synthesis of neurotransmitters, physical exercise stimulates increased levels of neurotrophins, such as the brain-derived neurotrophic factor (BDNF), insulin-like growth factor (IGF-1) and endothelialvascular growth factor (VEGF). Through increased synthesis and release of these growth factors, there is an improvement in cerebral neuroplasticity through neurogenesis, synaptogenesis and angiogenesis^23^^,^^24^^,^^25^. It is important to remember that in elderly people with AD, there may be a decrease in serotonergic fibers, which causes the presence of depressive symptoms. In this regard, BDNF can help compensate for this decline, as it acts to neutralize processes that are related to neurodegeneration and responsible for losses of neurons and serotonergic fibers. Thus, a relationship between the serotonergic system and BDNF has been suggested, implying that development of depressive symptoms may be associated with a decrease in BDNF^26^. In addition, BDNF favors regulation of brain plasticity in neural networks, which play important roles in depression. Thus, the contribution of BDNF to prevention of late-onset depression, which is directly associated with the etiology of AD, can be highlighted^26^. Another suggestive association that has been revealed is the improvement in mood and anxiety related to increased levels of IGF1^27^. Thus, growth factors can have a protective effect on NPS.

As shown in the present systematic review, all interventions through aerobic exercise were successful in relation to NPS. Therefore, it is interesting to highlight some discoveries about aerobic exercise through which patients with AD are likely to benefit regarding NPS.

A study by Sobol et al.^28^ demonstrated an increase in the peak oxygen uptake in patients with mild AD in association with NPS, through an intervention using aerobic exercise. On the other hand, other studies have already shown that this type of training improves blood flow and cerebral oxygenation, and contributes towards increasing the volume of the hippocampus, which is related to higher serum levels of BDNF, thus improving memory. This may help to mitigate NPS^29^. As can be seen, one of the etiological paths for NPS is a chain of causality, i.e. the behavioral symptom reflects the cerebral state of cognition^30^^,^^31^. Furthermore, it is pertinent to point out that it has been shown that people with depression have a reduced hippocampal volume, which is significantly important for these patients^32^.

In addition to the line of research that maintains that there is a close relationship between cognitive impairment and severe NPS^33^, a study on the action of aerobic exercise was conducted using an experimental model with AD in which swimming was the exercise. Increased levels of irisin, i.e. myokine, were secreted by skeletal muscles in response to physical movement, expressed in the hippocampus. Irisin stimulates higher levels of BDNF in the hippocampus, which activates neurogenesis and synaptogenesis paths^34^. Increased irisin levels have been seen to provide benefits regarding the synaptic plasticity of experimental models with AD^34^. Hence, irisin can also indirectly promote improvement of behavioral symptoms.

It is also worth noting the action of aerobic exercise on the neuroendocrine system. This may be the mechanism through which this training possibly favors reduction of NPS in AD. The neuroendocrine system is responsible for homeostatic balance and is affected by neuronal loss, although there has been little discussion of this. One of the important neuroendocrine axes when dealing with AD is the hypothalamic-pituitary-adrenal (HPA) axis, which is especially related to regulation of the stress response. It has been suggested that activity on the HPA axis is higher in AD cases, and this has been attributed to elevated cortisol levels in the cerebrospinal fluid, urine and serum of patients with AD. This leads to neuropsychiatric disorders^26^^,^^35^. In this regard, physical exercise helps to regulate the HPA axis, and this has been confirmed by studies that revealed significant reductions in cortisol in response to aerobic training^36^.

### Implications of the effectiveness of physical exercise on the neuropsychiatric symptoms of Alzheimer's disease

Based on improvement of the NPS of elderly people with AD, studies have indicated that there is a reduction in caregiver stress burden^37^^,^^38^. On the other hand, caregivers’ burdens give rise to changes in their lives, with reductions in intimacy and in the social cycle. Their burdens area stressful, depressing and frustrating experience that results in losses both to their health and to the quality of care that they provide for the elderly person with AD^39^^,^^40^. This is alarming from a social point of view, considering that sick caregivers are unable to correctly provide assistance to elderly people with AD. Such situations are reflected in increased need for public assistance and institutionalization. In addition, worsening of NPS has also been associated with increased risk of mortality^38^. For these reasons, it is observed that mitigation of NPS provides lower rates of institutionalization, lower spending on diseases and decreased mortality risks. Thus, exercise constitutes a valuable proposal for avoiding and minimizing these possible problems^41^.

Another important implication of physical exercise for NPS in AD is the positive association between cognitive performance and the clinical condition of NPS^17^. Worsening of NPS can aggravate the overall cognitive functioning but, fortunately, the opposite is also true: improvement of NPS favors functioning of cognition^13^. Moreover, diminution of NPS through physical exercise has been found to provide benefits regarding functional capacity, for carrying out activities of daily living, and improved quality of life among elderly people with AD^11^^,^^13^. NPS have been shown to have a direct relationship with performing functional activities, since systematized physical activity reduces NPS and has a positive effect on the functionality of elderly people with AD, thereby contributing to an improvement in these patients’ quality of life^13^.

On the other hand, it is extremely important to emphasize that besides these benefits, practicing physical exercise favors reduction of NPS such that health is boosted in an overall manner, through development of muscle endurance, cardiovascular capacity, joint mobility, motor coordination and balance. Thus, metabolic diseases, among others, can be prevented. Such benefits can only be achieved through practicing physical exercise.

This systematic review indicates that physical exercise can be a favorable non-pharmacological means for attenuating NPS among elderly people with AD, especially aerobic exercise. Moreover, physical exercise predisposes towards several neurobiological processes for this purpose. In this regard, physical exercise favors reduction of the severity and frequency of NPS in AD, with positive effects on cognitive and motor aspects of AD. Consequently, exercise can contribute towards postponing the evolution of this disease between its phases, thereby enabling significant improvements in the prognosis for the disease. Therefore, it is essential to highlight that the worsening of AD that is seen from one stage to the next needs to be taken into consideration in prescribing exercises, in order to maximize their benefits. Furthermore, it is important to explain some parameters that were demonstrated in the studies of this review, which gave rise to positive results regarding the effectiveness of physical exercise for diminishing NPS, in addition to the recommendations for AD that are set forth in the current literature. These parameters include prescription of moderate-intensity exercises, a minimum frequency of application of the exercises of three times a week and session duration of 45 to 60 minutes. Additionally, adherence to practicing physical exercises over a minimum time of three months is highlighted, in order to demonstrate benefits regarding NPS. as also are the positive effects of physical activity performed in groups. However, it is extremely important to emphasize that there is a need for further research to investigate the variables of physical exercise in AD in greater depth.

These physical exercises not only benefit the elderly individual with AD, but also aid their family members or caregivers. This further increases the scope and social importance of contributing to improvement of NPS that arise from AD. However, further studies in which NPS are their main outcome are needed, along with research to investigate the effects of different types of protocols on NPS in AD, such as experimental studies comparing the effects of physical exercise in isolation and use of a dual task protocol. Furthermore, some research to as certain how long the adherence to physical exercise needs to be, for improvements in NPS in AD to be seen, would be important. Lastly, there need to be more studies to investigate the pathological pathways through which NPS come to be present; the relationships between cognitive symptoms and NPS; and the neurobiological processes of physical exercise that become effective in NPS.
